# Impact of pharmacy services on initial clinical outcomes and medication adherence among veterans with uncontrolled diabetes

**DOI:** 10.1186/s12913-018-3665-x

**Published:** 2018-11-14

**Authors:** Justin D. Gatwood, Marie Chisholm-Burns, Robert Davis, Fridtjof Thomas, Praveen Potukuchi, Adriana Hung, M. Shawn McFarland, Csaba P. Kovesdy

**Affiliations:** 1 College of Pharmacy, University of Tennessee Health Science Center, Memphis, TN USA; 2 College of Medicine, University of Tennessee Health Science Center, Memphis, TN USA; 3 Memphis VA Medical Center, Memphis, TN USA; 4 Vanderbilt University School of Medicine, 1161 21st Avenue South, S-3223 Medical Center North Nashville, Nashville, TN 37232 USA; 5 Nashville VA Medical Center, Nashville, TN USA

**Keywords:** Adherence, Veterans, Diabetes, Disease management, Pharmacy services

## Abstract

**Background:**

Diabetes remains a growing public health threat but evidence supports the role that pharmacists can play in improving diabetes medication use and outcomes. To improve the quality of care, the Veterans Health Administration has widely adopted care models that integrate clinical pharmacists, but more data are needed to interpret the impact of these services. Our objective was to assess clinical pharmacy services’ impact on outcomes and oral antidiabetic medication (OAD) use among veterans with uncontrolled diabetes in the first year of therapy.

**Methods:**

This was a retrospective cohort analysis using the Veterans Affairs (VA) Corporate Data Warehouse to identify the first diagnosis of and initiation of OAD therapy for uncomplicated, uncontrolled diabetes (A1C > 7.0%) during 2002–2014. Receipt of clinical pharmacy services was identified using codes within VA electronic health records, and clinical values were obtained at or near the initial fill date and 365 days later. Use of OADs was assessed by proportion of days covered (PDC) for one year following the first filled prescription. Veterans having received clinical pharmacy services were matched 1:1 to those having not seen a clinical pharmacist in the first year of therapy, and generalized linear models assessed changes and differences in outcomes.

****Result**s:**

The analysis included 5749 patients in each cohort. On average, patients saw a clinical pharmacist 2.5 times throughout the first year of OAD therapy. Adherence to OAD medications was higher in veterans having seen a pharmacist (84.3% vs. 82.4%, *p* < 0.0001) and more such patients achieved a PDC of at least 80% (72.2% vs. 68.2%, *p* < 0.0001). After one year of OAD therapy, mean change in hemoglobin A1C was greater among those receiving pharmacy services (− 1.5% vs. -1.4%, *p* < 0.0001).

**Conclusion:**

Pharmacist participation in diabetes patients’ primary care positively affects the multifaceted needs of patients with this condition and comorbid chronic disease.

**Electronic supplementary material:**

The online version of this article (10.1186/s12913-018-3665-x) contains supplementary material, which is available to authorized users.

## Background

While much of the focus of the Affordable Care Act (ACA) has been on its improving access to care for millions of Americans, this legislation also had a profound effect on how pharmacists could be leveraged as members of primary care teams. Specifically, provisions of the ACA facilitated pharmacist incorporation with patient primary care through integrated and collaborative models of care and expanded opportunities to provide medication therapy management (MTM) services in conjunction with changes to Medicare Part D and within the scope of Accountable Care Organizations (ACO) [1]. Consequently, and in concert with the growth in patient-centered medical home (PCMH) models across the United States, pharmacists are now more intimately involved with direct patient care than ever before [2].

Providing support for such opportunities, a growing body of evidence has demonstrated the impact that clinical pharmacist involvement can have on outcomes in primary care settings, particularly among diabetes patients. Within this population, the vast majority of investigations demonstrated significant hemoglobin A1C reduction among patients having visited or received services from a clinical pharmacist [3–32]. Moreover, significantly more patients achieved recommended American Diabetes Association (ADA) treatment goals and, while less consistent, those having seen a clinical pharmacist often realized significant, positive changes in other clinical measures, such as systolic or diastolic blood pressure, low-density lipoprotein (LDL) levels, and body mass index (BMI) [4, 7–10, 12–14, 18, 19, 21, 32–37]. Importantly, positive results have been observed in diverse patient populations from all corners of the United States and included Latinos, those from underserved areas, high-risk patients, rural residents, those nonadherent to medications, and members of health maintenance organizations as well as current and former members of the armed forces [3, 4, 8, 9, 12–14, 34, 37]. Studies also reported that adding pharmacists to primary care teams is a cost-effective risk reduction strategy that can lead to significant cost avoidance among patients utilizing these services [7, 20–22, 38–40].

While results generally demonstrated that clinical pharmacy services have a positive impact on outcomes in patients with diabetes, these analyses tended to be limited by their sample size, length of follow-up, lack of control group, the inclusion of only 1 or a few sites, and variability in the point in time at which patients were intervened following either their diabetes diagnosis or treatment initiation [3–7, 9, 12, 13, 17, 20–22, 25–32, 34–36]. Recognizing these shortcomings, this study sought to evaluate the impact of clinical pharmacist services as implemented across a nationwide health system using a more robust and longitudinal data source: the Veterans Affairs (VA) electronic health record (EHR) system. Such a system is unique in its combined closed nature, nationwide data capture, and ability to identify each patient’s initial diagnosis as well as his or her treatment trajectory through the VA system. Moreover, since the VA has been particularly proactive in its adoption of clinical pharmacy services as part of their primary care teams their EHR includes codes specifically used to identify visits with clinical pharmacists. Consequently, in contrast to previously published work on pharmacist visits, the current study provides a broader examination of the pharmacist’s role as a resource to patients throughout a nationwide health system. Specifically, this research examines the initial impact of clinical pharmacy services among veterans with newly diagnosed, uncontrolled diabetes during their first year of oral antidiabetic (OAD) therapy.

## Methods

### Study design and data source

This was a retrospective observational matched cohort study that used the VA Corporate Data Warehouse from 2002 through 2014 and included extracts from the VA Decision Support System National Data Extracts, Medical SAS Datasets, and the Vital Status Files [41]. This study was approved by institutional review boards at the University of Tennessee Health Science Center and the Memphis VA Medical Center.

**Fig. 1:**
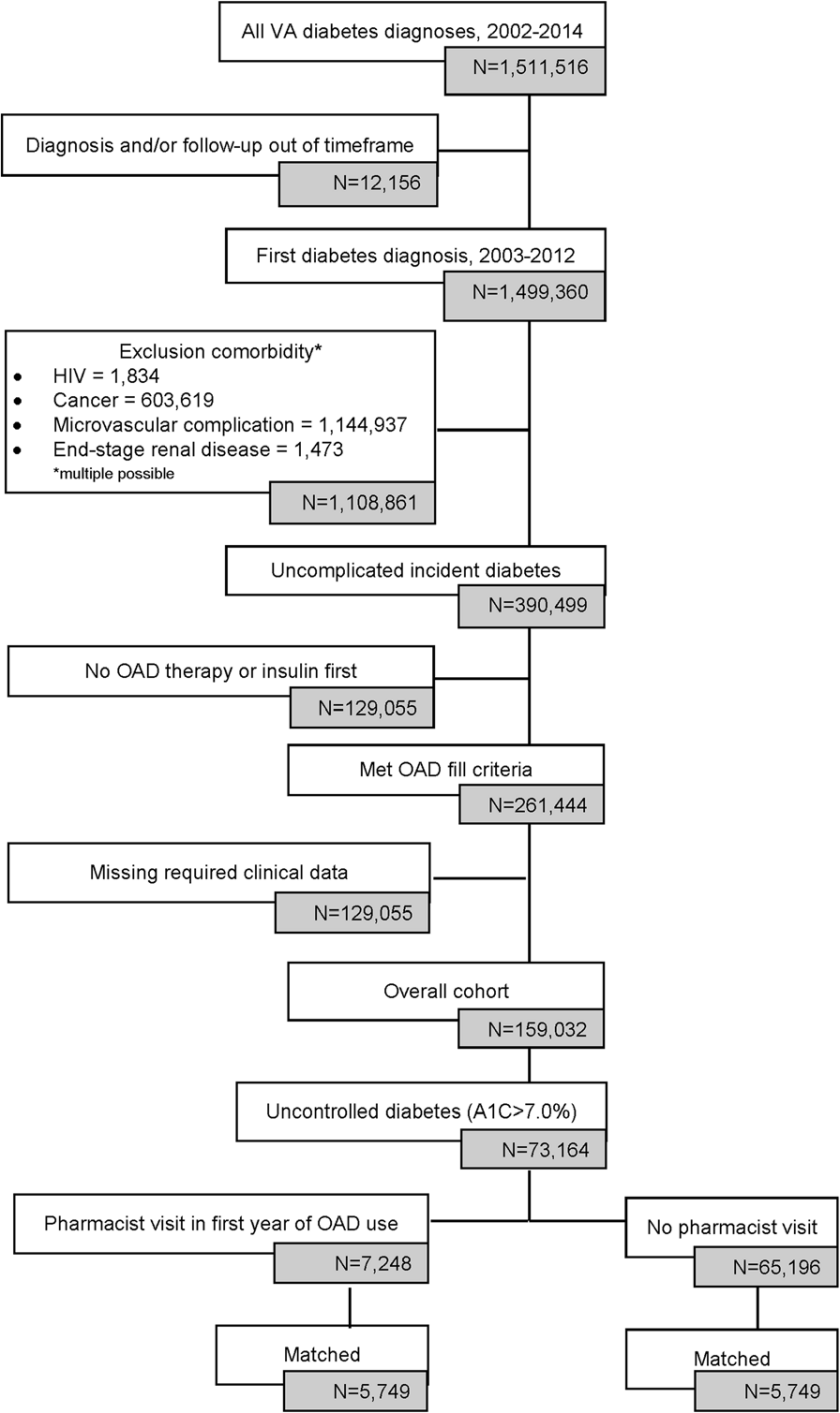
Patient Selection

The current analysis included a subset of a larger cohort that included incident cases of veterans diagnosed with uncomplicated diabetes mellitus (DM) between January 1, 2003 and December 31, 2012. Those included in the overall cohort must have been at least 18 years of age, diagnosed with diabetes without complications (ICD-9-CD codes: 250.00 or 250.02) for the first time (i.e., no diagnostic codes indicating DM in the year prior to the initial diagnosis), and prescribed an oral antidiabetic drug (OAD) as their first-line therapy for the first time (i.e., no OAD fills in the year prior to the first DM diagnosis). Additionally, patients must have had at least 1 year of data prior to and 2 years of data following their initial OAD prescription. Exclusion criteria included evidence of insulin dependence (according to ICD-9-CD codes and pharmacy records), having been diagnosed with a diabetes-related microvascular complication prior to or in conjunction with their initial DM diagnosis (see Additional file 1 for codes), having been diagnosed with HIV at any point, or having been diagnosed with malignant cancer prior to the initial, qualifying DM diagnosis. The overall study cohort included 159,032 patients (Fig. 1).

To address the current research questions, the study cohort was queried for service codes within the VA EHR used to indicate an outpatient visit to a clinical pharmacist. Separate codes are recorded within the VA for visits that were either face-to-face or by telehealth mechanisms; however, for purposes of this analysis having at least 1 of either type of visit in the first year since starting OAD therapy qualified a patient for inclusion. Additionally, patients needed to have a baseline (+/− 90 days of the initiation of OAD therapy) hemoglobin A1C value indicating uncontrolled DM (hemoglobin A1C > 7.0%) and have another test recorded 1 year later (+/− 90 days) [42].

### Pharmacy services

To improve the delivery of primary care services, the VA designed patient-aligned care teams in line with principles of the patient-centered medical home model. These teams are broken down into 2 components: the core team and the expanded team. The core team is comprised of the patient, a physician, a physician’s assistant or nurse practitioner primary care professional, a registered nurse care manager, a clinical staff assistant, and a medical administrative staff member. To provide more specialized care, the expanded team involves the core team members as well as clinical specialists, which includes a clinical pharmacy specialist (CPS), nutritionist, social worker, and mental health clinicians. Patients are referred to the CPS by the patient’s primary care physician when they require more specialized comprehensive medication management and can also be identified utilizing VA-based population management tools [43]. A CPS has an expanded scope of practice within the VA, involving them in direct patient care roles, including those focused on inpatient care, primary care, mental health services, and pain, and granting them advanced practice provider status, which authorizes them to perform medication optimization services with prescriptive authority. Within patient-aligned care teams, the CPS provides direct patient care through comprehensive medication management to start, change, or discontinue treatment and contributes to disease management in high-volume areas, such as diabetes.

### Outcomes

Patients were evaluated for multiple outcomes in the first year following initiation of OAD therapy, the primary outcomes of which included adherence to OAD therapy, changes in hemoglobin A1C, and achieving the recommended hemoglobin A1C target [42]. Adherence to OAD therapy was assessed over the first year of treatment by using refill records to calculate the proportion of days covered (PDC) [44]. The numerator for this metric used a sum of the days supply within each patient’s records to determine the amount of medication on hand while adjusting for overlapping days between fills; the denominator was the number of days in the first year of therapy following the initial fill (365). Similar to other studies involving adherence to common chronic medications, a PDC threshold of 80% was the cutoff for being adherent to therapy [45].

Secondary outcomes included changes from baseline in serum cholesterol, systolic and diastolic blood pressure, and weight, adherence to antihypertensive and lipid-lowering drugs, and achieving recommended blood pressure goals. Similar to hemoglobin A1C, clinical measures were evaluated at or near (+/− 90 days) OAD treatment initiation and then again 1 year later (+/− 90 days). Adherence to antihypertensives and lipid-lowering drugs was calculated over the first year of OAD therapy and compared between cohorts using the same calculation method employed for OADs. The extent to which recommended blood pressure treatment goals were attained (systolic <140mmHG, diastolic < 90 mmHg) was measured at both baseline and then 1 year later [46]. Changes in blood pressure and serum cholesterol levels and goal attainment were calculated only among those having been prescribed an antihypertensive or lipid-lowering agent prior to or during the observation year.

### Statistical analyses

Following identification of patients with at least 1 clinical pharmacist visit in the first year of OAD therapy, a 1:1 propensity score match was conducted using a caliper width of 0.2 of the standard deviation of the logit of the propensity score. Baseline covariates used to balance the cohorts included: age at OAD initiation, race, region of the country, gender, initial medication, specific comorbid conditions (cerebrovascular disease, myocardial infarction, congestive heart failure, chronic obstructive pulmonary disorder, peripheral artery disease), body mass index, and hemoglobin A1C. Standardized differences were calculated to assess the balance of the match where a difference of 0.1 or less indicated a good match [47, 48]. Tests of proportions (chi square and McNemar’s tests) and Wilcoxon rank sum tests were used to compare medication adherence values, changes in clinical values, and goal attainment between and/or within groups. SAS Enterprise Guide (SAS, Cary, NC) was used for all analyses.

## Results

### Study population

Out of the original study cohort of 159,032, 13,951 veterans were identified as having at least 1 clinical pharmacist visit in the first year following OAD initiation; ultimately, 5749 were successfully matched to veterans with incident diabetes without either type of pharmacist visit (Figure 1). Standardized differences indicated that the match provided good balance for the analysis (Table 1). The resulting sample was nearly all male (96.2%), mostly (70.7%) 55 years of age or older and white (76.9%) with a large proportion residing in the southern part of the United States (43.9%).

**Table 1:** Patient Characteristics

Characteristic	Pharmacist Visit N (%)	No Pharmacist Visit N (%)	Standardized Differences
Count	5749	5749	
Age			
< 35	56 (1.1)	56 (0.97)	−0.02
35–44	346 (6.02)	426 (7.4)	−0.06
45–54	1138 (19.8)	1339 (23.3)	−0.09
55–64	2537 (44.1)	2453 (42.7)	0.03
65–74	1116 (19.4)	982 (17.1)	0.06
75–84	488 (8.5)	423 (7.4)	0.04
85+	68 (1.2)	61 (1.1)	0.01
Race			
White	4374 (76.1)	4468 (77.7)	−0.04
African American	1182 (20.6)	1077 (18.7)	0.05
Asian	60 (1.0)	56 (1.0)	0.01
Native American	54 (0.9)	68 (1.2)	−0.02
Hawaiian/Pacific Islander	79 (1.4)	80 (1.4)	−0.002
Geographic Region			
Northeast	475 (8.3)	519 (9)	−0.03
Midwest	1484 (25.8)	1372 (23.9)	0.05
South	2479 (43.1)	2565 (44.6)	−0.03
West	1311 (22.8)	1293 (22.5)	0.01
Male	5535 (96.3)	5526 (96.2)	0.003
Initial Medication			
Metformin	3677 (63.9)	3610 (62.8)	0.02
Sulfonylurea	1903 (33.1)	1950 (33.9)	−0.02
Thiazolidinedione	74 (1.3)	87 (1.5)	−0.02
All other OADs	95 (1.7)	102 (1.8)	−0.01
Charlson Comobidity Index^a^	0.5 (0.68)	0.5 (0.65)	−0.05
Previous Event/Condition			
Cerebrovascular Disease	354 (6.2)	337 (5.9)	0.01
Myocardial Infarction	302 (5.3)	259 (4.5)	0.03
Congestive Heart Failure	313 (5.4)	283 (4.9)	0.02
Chronic Pulmonary Obstructive Disease	1237 (21.5)	1189 (20.7)	0.02
Peripheral Artery Disease	261 (4.5)	284 (4.9)	−0.02
Body Mass Index^a^	33.5 (6.0)	33.4 (5.8)	0.01
Baseline Hemoglobin A1C, %^a^	8.6 (1.6)	8.7 (1.6)	−0.04

^a^Values listed are mean (SD)

Additionally, about two-thirds of all patients started OAD therapy on metformin and the majority did not have another significant comorbidity; however, chronic obstructive pulmonary disease (COPD) was evident in more than 20% of those included.

On average veterans with diabetes who visited a clinical pharmacist did so 2.5 times (SD: 2.4; median: 2) in the first year after beginning OAD therapy, and their first visit took place, on average, within 4 months of treatment initiation (mean: 115.4 days; SD: 108.8). The vast majority of recorded visits (89.0%) were face-to-face with a pharmacist; only 8.0% of included veterans had a visit over the phone.

### Medication use

Across the study population, adherence to OAD therapy averaged 83.4% (SD: 23.5). Adherence was highest among patients on a thiazolidinedione (87.8% [SD: 23.5]) and was weakly correlated with hemoglobin A1C at treatment initiation (ρ = 0.15, *p* < 0.0001). Additionally, a higher proportion of patients (2.4% more) who saw a pharmacist either changed OAD therapy or added another oral medication to their regimen in the first year (*p* = 0.008). Those veterans with diabetes having visited a clinical pharmacist had slightly better adherence (*p* < 0.0001) over the first year compared to those without such a visit: 84.3% (SD: 22.9) versus 82.4% (SD: 24.1). Also, a higher proportion of veterans (*p* < 0.0001) with at least 1 clinical pharmacist visit in the first year were considered adherent (PDC ≥ 80.0%): 72.2% versus 68.2% (Table 2).

**Table 2:** Proportion Adherent to Oral Antidiabetic, Antihypertensive, and Lipid-Lowering Therapies in the First Year of Antidiabetic Treatment

Medication Class	Percent Adherent		*p*-value
	Pharmacist Visit	No Pharmacist Visit	
Oral Antidiabetic	72.2	68.2	< 0.0001
Antihypertensive	83.6	80.1	< 0.0001
Lipid-Lowering	59.1	53.9	< 0.0001
Oral Antidiabetic and Antihypertensive	64.6	60.9	< 0.001
Oral Antidiabetic and Lipid-Lowering	49.9	44.8	< 0.0001
Oral Antidiabetic, Antihypertensive, and Lipid-Lowering	46.9	42.5	< 0.0001

Patients on each therapy (Pharmacist Visit/No Visit): OAD (5749/5749),

Antihypertensive (5039/4892), Lipid-Lowering (4644/4417),

OAD, Antihypertensive, and Lipid-Lowering (4188/3900)

Adherence determined by achieving a PDC ≥80%

Antihypertensive and lipid-lowering agent use differed by treatment group as well. By the time an OAD was initiated, 69.9% of included patients were on at least 1 antihypertensive while 55.8% were taking a lipid-lowering drug. Over the first year of OAD use, adherence to antihypertensives and lipid-lowering agents was slightly higher among veterans who had a clinical pharmacist visit: 85.1% (SD: 22.2) versus 83.7% (SD: 23.0) and 78.6% (SD: 24.4) versus 75.6% (SD: 26.4), respectively (both *p* < 0.0001). Additionally, a higher proportion of veterans with a pharmacist visit achieved a PDC of at least 80% (Table 2) for both of these drug classes (both *p* < 0.0001): 83.6% versus 80.1% (antihypertensive) and 59.1% versus 53.9% (lipid-lowering drugs).

Among veterans on an OAD and a concomitant antihypertensive and/or lipid-lowering agent, adherence levels between OADs and these other drug classes were moderately correlated: ρ = 0.22 for antihypertensives (*p* < 0.0001) and ρ = 0.34 for lipid-lowering drugs (*p* < 0.0001). Between groups, a higher proportion of veterans were adherent to both their OAD and antihypertensive if they had a pharmacist visit during the year (Table 2): 64.6% versus 60.9% (*p* < 0.001). Similarly, more veterans on a lipid-lowering drug were adherent to both that medication and their OAD if they had a pharmacist visit: 49.9% versus 44.8% (*p* < 0.0001). This was also true of patients on all 3 medication classes as a higher proportion of those having seen a clinical pharmacist were adherent to all 3 classes in the first year: 46.9% versus 42.5% (*p* < 0.0001).

### Clinical measures

At treatment initiation, hemoglobin A1C was marginally different between groups (0.06%), averaging 8.6 (SD: 1.60) and 8.66 (SD: 1.63) for veterans who did and did not have a clinical pharmacist visit in the following year, respectively (*p* = 0.027). No statistically significant differences in systolic blood pressure (SBP), diastolic blood pressure (DBP), low-density lipoprotein (LDL), and high-density lipoprotein (HDL) were observed at baseline (respective *p*-values: 0.874, 0.477, 0.551, 0.694); however, slight differences in baseline weight (1.0 kg) were evident (*p* = 0.03).

Mean change in hemoglobin A1C from baseline across all patients was − 1.4% (SD: 1.95) with those adherent to their OAD therapy experiencing a significantly larger reduction over the first year (− 1.5% [SD: 1.91] versus − 1.4% [SD: 1.99], *p* < 0.0001). At the end of the first year, average hemoglobin A1C was 0.2% lower among veterans who had a clinical pharmacist visit (*p* < 0.0001). By the end of the first year of OAD use, 54.1% of those who visited a pharmacist achieved an A1C < 7% versus 48.9% for those without a visit (*p* < 0.001).

Improvements in the other clinical measures were also observed in the first year of OAD therapy, both within and between cohorts (Table 3). Systolic and diastolic blood pressure declined significantly in both groups (all *p* < 0.0001), with statistically similar changes in both SBP (*p* = 0.161) and DBP between groups (*p* = 0.836). Relatively weak correlations were observed between level of adherence and change in blood pressure over the course of the observation year (systolic: ρ = − 0.04, *p* < 0.0001; diastolic: ρ = − 0.05, *p* < 0.0001). However, significant differences in blood pressure reduction were observed among veterans adherent to their antihypertensive compared to those who were nonadherent: systolic pressure dropped 3.0 mmHg in the adherent group versus 0.9 mmHg in the nonadherent group and diastolic pressure dropped 2.5 mmHg in the adherent group compared with 1.1 mmHg in the nonadherent group (both *p* < 0.0001). Over the first year of concomitant OAD and antihypertensive therapy, the proportion of veterans achieving blood pressure goals increased irrespective of cohort, and no statistically significant differences in the change in proportions were observed between groups in reaching systolic, diastolic, or both blood pressure goals (respective *p*-values: 0.466, 0.428, 0.438).

**Table 3:** Baseline and One-Year Clinical Values by Pharmacist Visit

Measure	Pharmacist Visit		No Pharmacist Visit		*p*-value*
	Baseline	One Year	Baseline	One Year	
Hemoglobin A1C, %	8.0 (7.4–9.3)	6.9 (6.4–7.6)	8.0 (7.4–9.5)	7.0 (6.4–7.8)	0.004
SBP, mmHg	134.0 (124–144)	131.0 (121–141)	135.0 (124–144)	132.0 (122–142)	0.161
DBP, mmHg	79.0 (72–86)	77.0 (70–84)	80.0 (72–86)	78.0 (70–84)	0.836
LDL, mg/dL	97.0 (75–124)	89.0 (71–111)	97.0 (77–123)	90.0 (71–114)	0.005
HDL, mg/dL	37.0 (31–43)	38.0 (32–44)	35.8 (30–42)	36.0 (31–43)	0.711
Weight, kg	102.5 (90.3–117.0)	101.6 (89.2–115.7)	103.0 (90.7–117.5)	101.8 (89.6–101.8)	0.618

Values listed are median (IQR)

*p-value for comparison of change in values

Statistically significant changes in HDL and LDL were observed in both groups (all *p* < 0.0001) with larger reductions in median LDL in the pharmacist visit group (− 8.0 mg/dL versus − 7.0 mg/dL, *p* = 0.005); changes in HDL levels were similar across groups (*p* = 0.711). Similar to results in blood pressure values, changes in LDL levels were only weakly correlated with level of adherence to lipid-lowering drugs (LDL: ρ = − 0.05, *p* < 0.0001) while no statistically significant relationship was observed for HDL levels (*p* = 0.640). Significantly larger reductions in mean LDL were realized among patients adherent to their lipid-lowering agent during the observation year (− 11.0 mg/dL versus − 7.2 mg/dL, *p* < 0.0001) but no statistically significant differences in HDL change were observed based on adherence status (*p* = 0.972).

## Discussion

Our results provide valuable insight on the extent to which visits with a clinical pharmacist can effectively contribute to managing the complex needs of veterans with diabetes in the first year of antidiabetic therapy. Compared to veterans with diabetes who had not seen a clinical pharmacist, those who had experienced significantly larger reductions in hemoglobin A1C over the first year of OAD therapy. Pharmacist visits were also associated with higher rates of OAD, antihypertensive, and lipid-lowering drug adherence, which is likely reflective of the benefit pharmacists provide in counseling patients on the importance of proper medication use. Although the observed differences in medication possession may not provide markedly higher clinical benefit, the achieving of higher rates of adherent patients (PDC ≥ 80%) may be clinically relevant as reaching this threshold has been associated with better health outcomes [49–51]. In addition to differences in medication use, veterans under the care of a clinical pharmacist had greater reductions in hemoglobin A1Cand LDL, both measures of critical importance in managing diabetes. Taken together, these results lend support to pharmacists effectively intervening patients with diabetes, and other related needs, in order to contribute to providing guideline-recommended care and achieving objectives set forth by the ADA. However, as the statistical significance of the results were driven by the sample size, real-world implications of the findings should consider the extent to which the observed changes in clinical measures indicate meaningful improvements in disease management.

Compared to previous studies of patients on oral diabetic therapy in the VA system, our results provide a more system-wide interpretation of clinical pharmacy services with at least comparable findings. Whereas prior studies observed mixed impact of pharmacists on clinical management and/or medication use in veterans with diabetes, the current analysis demonstrated more positive improvements in hemoglobin A1C and LDL cholesterol and higher rates of adherence [25, 30, 34, 37, 52–54]. However, it is important to note that several of these earlier studies were conducted prior to full implementation of PCMH models by the VA, potentially limiting pharmacist impact. As our analysis includes multiple years of data following widespread PCMH adoption, the current assessment may be a better representation of what clinical pharmacist incorporation may contribute to patient outcomes. Moreover, a major strength of this assessment is the sample size used as many studies examining clinical pharmacist impact are hindered by small cohorts. By using a large, nationwide records system we add insight from all portions of the country and do so among a larger collection of those receiving clinical pharmacy services.

This study was limited in several ways. First, the analysis was limited to patients within the VA system, which facilitates access to care for veterans across the country; therefore, results may not be generalizable beyond veterans with diabetes or to populations where access to care remains problematic. Secondly, the actual services provided by pharmacists could not be gleaned from electronic records, which limits our ability to interpret what methods may have been more or less effective across VA facility. Our analysis was also restricted to using data collected within VA facilities; consequently, patient medication use and other clinical data captured from non-VA facilities were missing. Moreover, data collected on patient clinical measures came straight from EHR sources and are subject to misclassification. Additionally, the sample included only those that had lab tests both at baseline and 1 year later; therefore, results include those who may be more inclined to be engaged with the healthcare system and their care, which may have led to better than average results. Finally, while PDC is a widely used and accepted measure of adherence, it is still an indirect method of describing medication use and may not be indicative of actual medication consumption.

In spite of these limitations, this study has important implications for management of patients with diabetes in primary care settings. By demonstrating that visits with clinical pharmacists can positively contribute to disease management, the results suggest a potential role for these practitioners to play. A recent report prepared for the Association of American Medical Colleges highlighted a looming crisis in the United States: in the next decade, a significant shortfall in practicing physicians is expected across the country, ranging from 61,700 to 94,700 [55]. Within primary care, this shortfall may exceed 30,000 by 2025, which would significantly affect Americans’ ability to access a wide range of necessary services [55]. As demand for primary care services continues to escalate, in part due to implications of the ACA, the US healthcare system needs to adapt innovative solutions to ensure that demand for services is being met. As primary care practices become increasingly challenged by demand, pharmacists may be able to step in and manage patients with diabetes once the focus of treatment involves oral or injectable therapies. Consequently, primary care physician time can then be freed to better accommodate an increasing patient base or address those with cases that are more complex.

Considering the extent to which the VA has implemented clinical pharmacy services across its facilities, this system remains an important source of evidence for the impact of pharmacist intervention. Future studies using VA data sources should examine the precise mechanisms by which pharmacists influence medication use and disease management, both in diabetes and other conditions, as well as the role that these practitioners may play in assisting with patients that have multiple chronic conditions and, therefore, more complex treatment regimens. Moreover, evaluations beyond those involving patients with uncomplicated diabetes can shed light on how pharmacists can affect health outcomes among those with more advanced disease or when multiple comorbid conditions are involved. Results may then provide timely guidance to non-VA systems as pharmacist provider status continues to grow and expanded collaborative practice agreements spread across the country. Finally, an additional consideration tied to involving clinical pharmacists are the costs of their services. A future analysis should involve determining the cost-benefit ratio of incorporating clinical pharmacists into the care of patients with diabetes and other chronic conditions to examine long-term economic impact, results of which will add to a growing body of analyses suggesting that pharmacist involvement is a cost-effective means to improve diabetes management [7, 21, 38, 39].

## Conclusions

The involvement of clinical pharmacists in the care of veterans with diabetes was associated with gains in hemoglobin A1C reduction and slightly better OAD use in the first year of therapy, rivaling the outcomes of patients receiving only physician-directed care. Health systems across the United States beyond the VA should further consider how clinical pharmacists could contribute to the management of patients with diabetes, particularly among those with potentially complex medication management needs.

## Additional file

Additional file 1: Diagnosis Codes. (PDF 8 KB)

## Abbreviations

ACA: Affordable Care Act
ACO: Accountable Care Organization
ADA: American Diabetes Association
BMI: Body Mass Index
COPD: Chronic Obstructive Pulmonary Disease
CPS: Clinical Pharmacy Specialist
DBP: Diastolic Blood Pressure
DM: Diabetes Mellitus
EHR: Electronic Health Record
HDL: High-Density Lipoprotein
LDL: Low-Density Lipoprotein
MTM: Medication Therapy Management
OAD: Oral Antidiabetic
PCMH: Patient-Centered Medical Home
PDC: Proportion of Days Covered
SBP: Systolic Blood Pressure
VA: Veterans Affairs
