# Supplementary material for: Impact of pharmacy services on initial clinical outcomes and medication adherence among veterans with uncontrolled diabetes
Source: BMC Health Serv Res. 2018 Nov 14;18:855. doi: 10.1186/s12913-018-3665-x (PMC6236984; doi:10.1186/s12913-018-3665-x)
Supplement: Supplementary file 1 — Diagnosis Codes. (PDF 8 KB) [file 12913_2018_3665_MOESM1_ESM.pdf]

Additional File 1: Diagnosis Codes

Diagnoses (ICD-9 CM)

HIV: 042-044.9, V08, 795.71
Malignant cancer: 140-172.9, 174-195.8, 200-208.9
Retinopathy: 250.5, 362.0x
Neuropathy: 250.6, 357.2
Foot ulcer: 707.1x
Nephropathy: 250.4x
End-stage renal disease: 585.6
Cerebrovascular disease: 430-438
Ischemic stroke: 434.x, 436.x
Transient ischemic attack: 435.x
Myocardial infarction: 410-410.9, 412
Peripheral arterial disease: 440.0-440.9, 443.x, 38.0, 38.1, 39.50, 39.22, 39.24-39.26, 29.28
Chronic obstructive pulmonary disease: 490-496, 500-505, 506.4
Congestive heart failure: 428-428.9
Hypoglycemia: 251.2, 250.8
